# Association of Night Eating with Depression and Depressive Symptoms in Korean Women

**DOI:** 10.3390/ijerph16234831

**Published:** 2019-12-01

**Authors:** Kyung Won Lee, Dayeon Shin

**Affiliations:** 1 Department of Food Science and Nutrition, Gwangju University, Gwangju 61743, Korea; kwlee@gwangju.ac.kr; 2 Department of Food and Nutrition, Inha University, Incheon 22212, Korea

**Keywords:** night eating, depression, depressive symptoms, nocturnal ingestion of food, Korea National Health and Nutrition Examination Survey

## Abstract

This study examined the associations of night eating with depression and depressive symptoms in Korean adults. The study used a nationally representative sample of 31,690 Korean adults (≥19 years old) from the Korea National Health and Nutrition Examination Survey from 2008 to 2013. The participants were divided into two groups based on status of night eating: night eaters (consuming ≥25% of total daily energy intake between 21:00 and 06:00) and non-night eaters. Depression was defined based on diagnosis by a doctor, whereas depressive symptoms were defined as feelings of sadness or desperation for more than two weeks in the last one year. Multivariable logistic regression analyses were performed to examine the relationship between night eating and odds of depression and depressive symptoms after controlling for age, education, income, marital status, drinking, smoking, day of recalled intake, physical activity, body mass index, menopausal status (women only), total energy intake, and sleep duration. A total of 14.3% of Korean adults were night eaters. Night eaters were more likely to be men, young, less educated, single, drinkers, current smokers, and not employed (all *p*s < 0.05). In women, night eaters had higher odds of depression (adjusted odds ratio [AOR], 1.33; 95% confidence interval [CI], 1.02–1.75; *p* for trend = 0.0389) and depressive symptoms (AOR, 1.20; 95% CI, 1.01–1.41; *p* for trend = 0.0382) compared with non-night eaters. However, no associations of night eating with depression and depressive symptoms were found in men. Night eaters had higher odds of depression and depressive symptoms only in Korean women. Future studies are warranted to elucidate the underlying psychological and behavioral mechanisms that in turn may shed light on the factors influencing both night eating and odds of depression and depressive symptoms.

## 1. Introduction

Depression is one of the most common mental health disorders, affecting more than 300 million people, or 4.4% of the global population [1]. According to the World Health Organization, depression ranks as the third cause of disease burden worldwide; it is projected to contribute most to the global burden of disease in 2030 [2]. In Korea, depressive disorders have emerged as the fourth leading cause of disability [3]. Given the growing socioeconomic burden of depression, many efforts have been made to identify the factors that can prevent or alleviate depression and depressive symptoms.

As a part of various efforts, researchers have sought to identify dietary factors related to depression. Night eating has been described through various expressions, such as night eating and nocturnal ingestion of food, characterized by a circadian delay in daily food ingestion [4,5,6]. Since the term “night eating syndrome (NES)” was first introduced by Stunkard et al. in 1955 [7], continuous attention has been given to the relationship between night eating and health outcomes. Depressive mood has been found to be more severe among individuals with NES, and prevalence of night eating increases in those with binge eating disorders [8], as well as schizophrenia [9]. A cohort study conducted by Colles et al. [10] found positive associations between NES, body mass index, and binge eating. They also reported that individuals having nocturnal snacking tend to have higher psychological distress [10].

Although studies have examined the relationship between night eating and psychiatric disorders, most of them have focused on night eating as one of the dietary behaviors in patients with psychological disturbance. For this reason, findings from previous studies are limited to patients with psychiatric disorders, and additional investigations focusing on the general population are needed.

Particularly, Korean society has distinct lifestyles, such as frequent overtime work and social gathering, as well as a late-night snacking culture. These lifestyles cause Koreans to consume food later than is usually thought to be dinner time. Given that depression is prevalent and the possibility of exposure to night eating is increased, studies exploring the relationship between night eating and psychiatric disorders among general Korean adult populations are needed. Therefore, we aimed to identify the various factors related to night eating habits and investigate the potential associations of night eating with the odds of depression and depressive symptoms using data from representative samples of the Korean adult population.

## 2. Materials and Methods

### 2.1. Data Source and Study Population

The Korea National Health and Nutrition Examination Survey (KNHANES) is an ongoing national surveillance system that was established to monitor the health, nutritional status, and prevalence of chronic diseases in the Korean population [11,12]. KNHANE uses a multi-stage clustered sampling design to obtain nationally representative samples of non-institutionalized Korean citizens, and consist of three surveys: health interview, health examination including physical examinations and clinical tests, and nutrition survey [13]. A total of 36,081 participants aged 19 years old and older completed the nutrition survey in KNHANES 2008–2013. For the present study, we excluded those who were reporting implausible dietary intake (<500 or >5000 kcal/day) (n = 570), pregnant and lactating women (n = 511), not eligible for data analyses because of missing responses on survey questionnaire items related to depression and depressive symptoms, and those with missing information on potential covariates (n = 3310). The final analytical sample of this cross-sectional study included 31,690 Korean adults (12,720 men and 18,970 women).

Informed consent was obtained from all participants, and all of the survey protocols and procedures were approved by the Korea Centers for Disease Control and Prevention (KCDC) Institutional Review Board [11,12].

### 2.2. Determination of Night Eating

We used the 24-h dietary recall data obtained from KNHANES to determine night eating status. During the 24-h dietary recall survey, participants were asked to report a clock time for each reported food or beverage item. The participants were classified into two groups based on the definition of night eating used in previous Korean studies [14,15,16]: night eaters (consuming ≥25% of total daily energy intake between 21:00 and 06:00) vs. non-night eaters. As a 24-h dietary recall that covered a 24-h time period (00:00 to 23:59) used in the KNHANES was adopted in this study, we defined “night (time)” as the combination of two time periods, 00:00 to 06:00 and 21:00 to 23:59, on the same calendar day.

### 2.3. Outcome Variables

Depressive symptoms were assessed by the following question: “In the last year, have you felt sadness or desperation for more than two weeks?”. Participants who answered “yes” were considered to have depressive symptoms. In addition, participants were asked if they had been diagnosed with depression by a doctor in their lifetime. Participants who answered “yes” were regarded as having depression.

### 2.4. Statistical Analyses

To account for the complex survey design, all analyses were weighted using the SURVEY procedure with sample weights, strata, and primary sampling units recommended by the KNHANES analytic guidelines [11]. All statistical analyses were performed using SAS (version 9.4; SAS Institute, Inc., Cary, NC, USA). Statistical significance was declared at *p* < 0.05.

The general and health-related characteristics, eating behavior, and nutrient intakes of participants were compared across the night eating status using the chi-squared test for categorical variables and multiple linear regressions for continuous variables. This study also used multiple logistic regression analyses to estimate the adjusted odds ratios (ORs) with 95% confidence intervals (CIs) after adjusting for potential covariates in three models. Model 1 was adjusted for age (years). Model 2 was additionally adjusted for education level (middle school graduates or less, high school graduates, and college graduation or higher), income level (lowest, lowest middle, upper middle, and highest), marital status (married or single), drinking (never/rarely, ≤1 times/month, >1 times/month), smoking (non-smokers, former smoker, current smoker), occupation (employed or unemployed), day of recalled intake (Mondays–Thursdays or Fridays–Saturdays), regular physical activity (yes or no), body mass index (kg/m^2^), and menopausal status (yes or no; women only). Model 3 was additionally adjusted for total energy intake (kcal/day) and sleep duration (hours).

## 3. Results

The general characteristics of the participants according to night eating habits are presented in Table 1. Among the 31,690 participants, 3504 (14.3%) were night eaters. Relative to non-night eaters, night eaters were more likely to be men, single, and current smokers, but less likely to be employed (all *p*s < 0.01). Night eaters also tended to consume alcohol more than one time per month (*p* < 0.05) and have shorter sleep duration (*p* < 0.01) compared with non-night eaters. However, income, day of recalled intake, and physical activity were not associated with night eating habits.

Eating behaviors according to night eating habits are presented in Table 2. Night eating habits were negatively associated with the main meal episodes but positively associated with snack episodes and length of eating period in both men and women. The number of main meal episodes and energy intake from main meals were significantly lower in night eaters compared with non-night eaters. In the contrast, night eaters reported higher number of total snack episodes and percentage of energy intake from night main meals, snacks, and night snacks (all *p*s < 0.01). In addition, time difference between the first and last eating episode differed according to night eating habits, with night eaters reporting a significantly longer ingestion period (*p* < 0.01). Compared with non-night eaters, a higher percentage of night eaters reported having breakfast, lunch, and all three main meals. The percentage of individuals having ≥25% of energy from snacks was more than two times higher in night eaters than non-night eaters.

Table 3 shows the total energy and nutrient intake by night eating habits. In both sexes, night eaters had a higher energy intake compared with non-night eaters (men: 2262 vs. 2139 kcal, *p* < 0.01; women: 1761 vs. 1641 kcal, *p* < 0.01). Night eating habits were positively associated with the percentage of energy from fat and sodium intake (all *p*s < 0.01). In contrast, night eaters showed lower intakes of carbohydrates, dietary fibers, potassium, calcium, and phosphorus (all *p*s < 0.01).

The multivariable-adjusted ORs for depression and depressive symptoms for night eaters and non-night eaters are listed in Table 4. In mode 3, the fully adjusted models, compared with non-night eaters, night eaters showed higher odds for depression (AOR, 1.33; 95% CI, 1.02–1.75; *p* for trend = 0.0389) and depressive symptoms (AOR, 1.20; 95% CI, 1.01–1.41; *p* for trend = 0.0382) only in women (Table 4).

## 4. Discussion

In this large cross-sectional study of free-living Korean adults, we explored the factors related night eating behaviors and the association of night eating status with depression and depressive symptoms. Our findings showed that 14.3% of Korean adults consumed more than 25% of their total daily energy intake between 21:00 and 06:00. Participants who were men, low educated, single, current smokers, not currently employed, and consumed alcohol ≥1 times/month were more likely to have a night eating habit compared with their counterpart (Table 1). This corresponded with the results from a previous study of Korean adolescents who participated in KNHANES [14]. Moreover, night eating habits were positively associated with depression and depressive symptoms. Interestingly, these associations were observed only in women, and remained statistically significant after controlling for potential covariates.

Earlier research has indicated that morning meals usually have carbohydrate-rich features, lunch meals are rich in protein, and evening meals have high fat contents [17]. In other words, evening meals show a high energy density compared with morning meals [18]. In addition, whereas early intake of the day is linked to overall decreases in food intake, intake at late evening increases overall food intake [18]. Similarly, in our study, night eaters reported a higher daily total energy intake and a higher percentage of energy from fat, as well as a lower percentage of energy from carbohydrates. Consistent with our findings, Lundgren et al. [19] reported that night eaters have more daily energy intake (night eaters: 2285 kcal vs. control: 1856 kcal) and nocturnal food ingestion compared with controls. They also reported that the time point when night eaters consume 75% of their daily total energy is four hours later compared with controls [19]. The type of foods eaten late at night tends to be unhealthy, palatable foods with high sugar, salt, and fat content, rather than healthy foods, such as fruits and vegetables [20,21]. Likewise, the results of our study indicated that night eaters had lower consumption of dietary fiber, potassium, calcium, and phosphorus, but higher consumption of fats and sodium compared with non-night eaters.

Epidemiological studies have investigated the associations of night eating or NES with mental health. Our results indicating positive relationships between night eating, depression, and depressive symptoms are in agreement with previous studies. A previous cross-sectional study of 404 Korean female nurses also found that NES is significantly associated with the increased odds of depressive symptoms [22]. A US study of overweight individuals reported that night eaters show distinct psychopathological patterns, such as greater depression and lower self-esteem. Another study, in which the two groups had no differences in amount of food intake, reported that night eaters feel less hunger and more full compared with non-night eaters [23]. Another cohort study of 431 Australian aged 18–71 years reported that participants with NES, who reported nocturnal snacking, tend to manifest higher symptoms of depression and hunger along with decreased mental health-related quality of life [10]. In our study, night eaters consumed approximately 20% of their daily total energy intake from night snacking, which was more than five times compared with non-night eaters. Combined with the previous findings, our results suggest that night eating and snacking might be linked to other psychological disturbances beyond depression and depressive symptoms.

The mechanisms underlying the relationships between night eating and depression and depressive symptoms remain unclear. Their relationships can be explained by three plausible pathways. First, night eating behaviors could contribute to the increased odds of depression or depressive symptoms. Particularly, night eaters are more likely to experience alterations in endogenous nocturnal hormones, namely, melatonin and leptin. According to Birketvedt et al. [4], night eaters have lower plasma melatonin and leptin levels, and higher levels of plasma cortisol relative to non-night eaters. Melatonin is a hormone that plays a role in inducing and maintaining sleep; lowered melatonin levels may lead to impaired sleep and depression [24]. In addition, the slowdown of the nocturnal increases in leptin among night eaters might lead to depression or depressive symptoms. Leptin, which helps suppress appetite and food intake and maintain sleep, tends to increase at night [25,26,27]. In our study, significant associations between night eating habits and odds of sleep duration were observed in women but not in men. In the fully adjusted model (model 3), night eaters were more likely to have a short sleep duration (≤6 h) (AOR, 1.20; 95% CI, 1.06–1.36; *p* for trend = 0.0043) compared with non-night eaters (Supplementary Table S1). Thus, night eating could contribute not only to depressive disorders but to insufficient or excessive sleep duration. Decreased nocturnal leptin levels owing to night eating can interfere with sleep or promote appetite, which in turn leads to a vicious cycle of night eating, impaired sleep, and depressive disorders. Second, night eating could be a means of expressing underlying depression or depressive symptoms. Orhan et al. (2011) demonstrated that prevalence of NES was higher in depressed patients compared to healthy control participants (35.2% vs. 19.2%) [28]. Other two studies from the United States reported that the higher risk for NES was observed among psychiatric clinic outpatients [29] and obese people with serious mental illness [30]. Third, common underlying factors such as insomnia might lead to both night eating behavior and depression or depressive symptoms. Rogers et al. (2006) indicated that insomnia or sleep disturbances precedes NES, and nocturnal eating which is a feature of NES causes sleep disturbances in people with NES [31]. In addition, another study of psychiatric outpatients reported that sleep disturbance was a significant predictor of NES [32]. We also found that individuals with short sleep duration (≤6 h) tended to have night eating compared with those with adequate sleep duration (6< to <9 h). Due to the nature of cross-sectional design, this study provided cross-sectional association between night eating and depression or depressive symptoms. Additional investigations using prospective cohort study are needed to determine whether night eating causes depression or vice versa.

To our knowledge, this study is the first to identify the associations of night eating status with depression and depressive symptoms in a representative sample of Korean adults. Therefore, results of the current study can be applicable to general Korean adult populations. In addition, we demonstrated that nocturnal ingestion of food could be a risk factor for depression and depressive symptoms in Korean women even after adjusting for various covariates.

The limitations of this study should also be considered. First, as KNHANES did not carry out a survey on NES, we defined night eating according to the time period of food intake or the percentage of energy consumed in a certain time period. Various definitions of night eating have been used in studies conducted in Western countries [33,34,35]. However, as people usually eat later in the day in Korea compared with the Westerners, this study defined night eating as a later time period than that used in Western studies. To date, a consensus on the operational definition of night eating has not been proposed; additional investigations using a NES questionnaire are warranted for a better understanding of the relationship between NES and depression in Korean populations. Second, KNHANES collected the information on depression and depressive symptoms by self-reports with simple questions. The definitions of depression and depressive symptoms used in the current work have been used widely to identify associations with dietary intakes and behaviors in previous studies of Koreans. However, to yield more accurate results, standardized tools, such as the Patient Health Questionnaire-9 [36], Beck Depression Inventory-II [37], and Center for Epidemiologic Studies Depression Scale [38], should be used in future investigations. Lastly, our cross-sectional analysis could not derive causal relationships of night eating status with depression and depressive symptoms. Further prospective cohort studies are needed to investigate these causal relationships among Korean adults.

## 5. Conclusions

Night eating was significantly associated with increased odds for depression and depressive symptoms in Korean women, but not in men. Several sociodemographic and health-related factors were related to night eating status. The results of this study could help establish dietary behavioral strategies for mental health. Future studies need to elucidate the underlying psychological and behavioral mechanisms that may link the factors influencing night eating and odds of depression and depressive symptoms.

## Figures and Tables

**Table 1:** General characteristics of study participants by night eating habits in Korean adults ^1^.

	Total	Non-Night Eaters	Night Eaters	*p* ^2^	Night Eating
	n (Wt’d %)	n (Wt’d %)	n (Wt’d %)		AOR (95% CI) ^3^
**Total**	31,690 (100.00) ^4^	28,186 (85.67)	3504 (14.33)		-
**Sex**				<0.0001 **	
Men	12,720 (49.44)	10,890 (47.20)	1830 (62.87)		1.00
Women	18,970 (50.56)	17,296 (52.80)	1674 (37.13)		1.23 (1.08–1.41) **
**Age (years)**	45.20 ± 0.17 ^5^	46.32 ± 0.18	38.51 ± 0.29	<0.0001 **	0.97 (0.96–0.97) **
**Education level**				<0.0001 **	
≤ Middle school	12,331 (28.64)	11,459 (30.36)	872 (18.36)		1.39 (1.19–1.63) **
High school	10,633 (39.46)	9150 (37.98)	1483 (48.29)		1.26 (1.14–1.40) **
≥ College	8726 (31.91)	7577 (31.67)	1149 (33.35)		1.00
**Income**				0.0780	
Lowest	7704 (25.27)	6877 (25.37)	827 (24.68)		1.00
Lower middle	8062 (25.65)	7142 (25.45)	920 (26.82)		1.12 (0.98–1.28)
Upper middle	7987 (24.84)	7057 (24.63)	930 (26.13)		1.14 (0.99–1.31)
Highest	7937 (24.24)	7110 (24.55)	827 (22.38)		1.01 (0.88–1.17)
**Marital status**				<0.0001 **	
Married	27,679 (78.80)	25,051 (81.05)	2628 (65.35)		1.00
Single	4011 (21.20)	3135 (18.95)	876 (34.65)		1.17 (1.02–1.34) *
**Drinking status**				< 0.0001 **	
Never/rarely	15,534 (42.09)	14,345 (44.36)	1189 (28.54)		1.00
≤1times/month	9833 (35.05)	8607 (34.60)	1226 (37.76)		1.08 (0.97–1.21)
>1 times/month	6323 (22.86)	5234 (21.04)	1089 (33.69)		1.62 (1.43–1.85) **
**Smoking status**				< 0.0001 **	
Never	19,384 (54.46)	17,685 (56.74)	1699 (40.83)		1.00
Former smoker	3978 (13.60)	3557 (13.75)	421 (12.67)		1.14 (0.96–1.35)
Current smoker	8328 (31.95)	6944 (29.51)	1384 (46.50)		1.50 (1.31–1.72) **
**Occupation**				< 0.0001 **	
Employed	13,391 (37.10)	12,450 (38.95)	941 (26.04)		1.48 (1.33–1.65) **
Not employed	18,222 (62.90)	15,672 (61.05)	2550 (73.96)		1.00
**Day of week of dietary intake**				0.1422	
Monday-Thursday	18,983 (59.66)	16,948 (59.92)	2035 (58.11)		1.00
Friday-Saturday	12,707 (40.34)	11,238 (40.08)	1469 (41.89)		1.06 (0.96–1.17)
**Regular physical activity ^6^**				0.0514	
Yes	15,887 (50.84)	14,081 (50.54)	1806 (52.68)		0.95 (0.87–1.04)
No	15,801 (49.16)	14,104 (49.46)	1697 (47.32)		1.00
**Body mass index (kg/m^2^)**	23.66 ± 0.03	23.69 ± 0.03	23.50 ± 0.08	0.0150 *	0.99 (0.97–0.99) *
**Sleep duration (h)**				0.4249	
≤6 (short)	1311 (40.30)	11,664 (40.26)	1469 (40.54)		1.14 (1.04–1.25) **
6 < to <9 (adequate)	16,064 (52.11)	14,305 (52.26)	1759 (51.24)		1.00
≥9 (long)	2493 (7.59)	2217 (7.48)	276 (8.22)		1.13 (0.95–1.35)

^1^ Data were from the Korea National Health and Nutrition Examination Surveys (KNHANES). All data were weighted to account for the complex study design according to the analytical guidelines of the KNHANES. ^2^ * *p* < 0.05, ** *p* < 0.01 ^3^ AOR, adjusted odds ratio; 95% CI, 95% confidence interval. Multiple logistic regression analysis was performed to estimate the odds for night eating for the study participants from the KNHANES 2008–2013: statistical model was adjusted for sex (men and women), age (continuous), education level (middle school graduates or less, high school graduates, and college graduation or higher), income (lowest, lowest middle, upper middle, and highest), marital status (married or single), drinking status (never/rarely, ≤1 times/month, and >1 times/month), smoking status (non-smokers, former smoker, or current smoker), occupation (employed or not employed), day of week of dietary intake (Monday–Thursday and Friday–Saturday), regular physical activity (yes or no), body mass index (continuous, kg/m^2^), menopausal status (yes or no, women only), and sleep duration (≤6, 6< to <9, or ≥9 h). ^4^ Values represented frequency (Wt’d %). ^5^ Mean ± SE ^6^ Having regular physical activity was defined as walking ≥5 time a week for ≥30 min each time.

**Table 2:** Eating behaviors by night eating habits in Korean adults ^1^.

	Men			Women		
	Non-Night Eaters	Night Eaters	*p* ^3^	Non-Night Eaters	Night Eaters	*p*
	(n = 10,890)	(n = 1830)		(n = 17,296)	(n = 1674)	
	**Mean ± SE**	**Mean ± SE**		**Mean ± SE**	**Mean ± SE**	
Number of total eating episodes ^2^	5.1 ± 0.1	5.1 ± 0.1	0.7007	5.0 ± 0.1	5.0 ± 0.1	0.8808
Number of total main meal episodes ^2^	2.7 ± 0.0	2.6 ± 0.0	<0.0001 **	2.5 ± 0.0	2.3 ± 0.0	<0.0001 **
Energy from main meals (%)	84.1 ± 0.3	73.5 ± 0.6	<0.0001 **	81.3 ± 0.4	71.7 ± 0.7	<0.0001 **
Energy from night main meals (%)	0.2 ± 0.1	23.1 ± 0.6	<0.0001 **	0.3 ± 0.2	23.4 ± 0.7	<0.0001 **
Number of total snack episodes ^2^	2.4 ± 0.1	2.5 ± 0.1	0.0092 **	2.4 ± 0.1	2.6 ± 0.1	0.0003 **
Energy from snacks (%)	15.9 ± 0.3	26.5 ± 0.6	<0.0001 **	18.7 ± 0.4	28.3 ± 0.7	<0.0001 **
Energy from night snacks (%)	3.3 ± 0.1	17.1 ± 0.5	<0.0001 **	3.4 ± 0.2	16.9 ± 0.6	<0.0001 **
Length of ingestion period (h) ^2^	12.0 ± 0.1	14.1 ± 0.2	<0.0001**	11.2 ± 0.1	13.1 ± 0.2	<0.0001 **
	**n (Wt’d %)**	**n (Wt’d %)**		**n (Wt’d %)**	**n (Wt’d %)**	
Reporting breakfast^2^	1578 (20.7)	567 (35.5)	<0.0001 **	2533 (18.1)	1108 (38.7)	<0.0001 **
Reporting lunch^2^	552 (5.7)	182 (11.2)	<0.0001 **	1173 (7.3)	209 (13.8)	<0.0001 **
Reporting dinner^2^	376 (3.9)	80 (4.8)	0.1424	1068 (6.8)	103 (7.0)	0.7495
Reporting all 3 main meals^2^	2343 (28.2)	733 (45.1)	<0.0001 **	4390 (29.3)	775 (51.2)	<0.0001 **
Reported ≥ 25% of energy from snacks	2327 (22.9)	924 (53.5)	<0.0001 **	4287 (26.5)	877 (55.3)	<0.0001 **

^1^ Data were from the Korea National Health and Nutrition Examination Surveys (KNHANES). All data were weighted to account for the complex study design according to the analytical guidelines of the KNHANES. The multivariable linear regression models included covariates including age (continuous), education level (middle school graduates or less, high school graduates, and college graduation or higher), income (lowest, lowest middle, upper middle, and highest), marital status (married or single), drinking status (never/rarely, ≤1 times/month, and >1 times/month), smoking status (non-smokers, former smoker, or current smoker), occupation (employed or not employed), day of week of dietary intake (Monday–Thursday and Friday–Saturday), regular physical activity (yes or no), body mass index (continuous, kg/m^2^), and menopausal status (yes or no, women only). ^2^ The models also included total daily energy (continuous) intake as an independent variable ^3^ *p* values were obtained from the multivariable linear regression analyses indicate the significance of the association of each independent variable with night eating habits (** *p* < 0.01).

**Table 3:** Total energy and nutrient intake by night eating habits in Korean adults ^1^.

		Men			Women		
		Non-Night Eaters	Night Eaters	*p* ^3^	Non-Night Eaters	Night Eaters	*p*
	2015 KDRIs ^4^	(n = 10,890)	(n = 1830)		(n = 17,296)	(n = 1674)	
		**Mean ± SE**	**Mean ± SE**		**Mean ± SE**	**Mean ± SE**	
Total energy, kcal		2139 ± 15	2262 ± 27	<0.0001 **	1641 ± 17	1761 ± 24	<0.0001 **
Carbohydrate, % of energy ^2^	55–65	63.7 ± 0.2	60.3 ± 0.3	<0.0001 **	68.9 ± 0.1	63.9 ± 0.4	<0.0001 **
Protein, % of energy ^2^	7–20	14.4 ± 0.2	14.2 ± 0.2	0.0860	14.1 ± 0.1	14.2 ± 0.2	0.6845
Fat, % of energy ^2^	15–30	17.9 ± 0.1	18.9 ± 0.2	<0.0001**	17.2 ± 0.1	19.5 ± 0.3	<0.0001 **
Dietary fiber, g^2^	M: 25, W: 21	8.1 ± 0.2	7.3 ± 0.2	<0.0001 **	7.0 ± 0.1	6.1 ± 0.1	<0.0001 **
Sodium, mg^2^	1100–1500	5249 ± 71	5612 ± 33.5	<0.0001 **	3761 ± 63	4040 ± 23	<0.0001 **
Potassium, mg^2^	3500	553.6 ± 3.6	517.4 ± 7.8	<0.0001 **	453.9 ± 2.7	417.1 ± 7.0	<0.0001 **
Calcium, mg^2^	700–800	3337 ± 14	3100 ± 31	<0.0001 **	1002 ± 2.6	944 ± 8	<0.0001 **
Phosphorus, mg^2^	700	1310 ± 4.0	1241 ± 9.2	<0.0001 **	2736 ± 12.8	2495 ± 32	<0.0001 **

^1^ Data were from the Korea National Health and Nutrition Examination Surveys (KNHANES). All data were weighted to account for the complex study design according to the analytical guidelines of the KNHANES. The multivariable linear regression models included covariates including age (continuous), education level (middle school graduates or less, high school graduates, and college graduation or higher), income (lowest, lowest middle, upper middle, and highest), marital status (married or single), drinking status (never/rarely, ≤1 times/month, and >1 times/month), smoking status (non-smokers, former smoker, or current smoker), occupation (employed or not employed), day of week of dietary intake (Monday–Thursday and Friday–Saturday), regular physical activity (yes or no), body mass index (continuous, kg/m^2^), and menopausal status (yes or no, women only). ^2^ The models also included total daily energy (continuous) intake as an independent variable ^3^ *p* values were obtained from the multivariable linear regression analyses indicate the significance of the association of each independent variable with night eating habits (** *p* < 0.01). ^4^ KDRIs, Dietary Reference Intakes for Koreans.

**Table 4:** The adjusted odds ratios (AORs) and 95% confidence intervals (CIs) for depression and depressive symptoms by night eating habits in Korean adults ^1^.

		Men			Women		
		Non-night Eaters	Night Eaters	*p* ^3^	Non-Night Eaters	Night Eaters	*p*
		(n = 10,890)	(n = 1830)		(n = 17,296)	(n = 1674)	
			**AOR (95% CI)**			**AOR (95% CI)**	
Depression ^2^	Model 1	1.00	0.82 (0.48–1.40)	0.4609	1.00	1.44 (1.10–1.88)	0.0077 **
	Model 2	1.00	0.82 (0.47–1.41)	0.4669	1.00	1.34 (1.02–1.76)	0.0360 *
	Model 3	1.00	0.81 (0.47–1.39)	0.4405	1.00	1.33 (1.02–1.75)	0.0389 *
Depressive symptoms	Model 1	1.00	1.06 (0.87–1.30)	0.5554	1.00	1.31 (1.11–1.55)	0.0015 **
	Model 2	1.00	1.01 (0.82–1.24)	0.9371	1.00	1.19 (1.01–1.40)	0.0476 *
	Model 3	1.00	1.01 (0.82–1.24)	0.9241	1.00	1.20 (1.01–1.41)	0.0382 *

^1^ Data were from the Korea National Health and Nutrition Examination Surveys (KNHANES). All data were weighted to account for the complex study design according to the analytical guidelines of the KNHANES. AOR, adjusted odds ratio; 95% CI, 95% confidence interval. Multiple logistic regression analysis was performed to estimate the odds ratio for depression and depressive symptoms for the study participants from the KNHANES 2008–2013, in three models: The Model 1 was adjusted for sex (men and women) and age (continuous); Model 2 was additionally adjusted for education level (middle school graduates or less, high school graduates, and college graduation or higher), income (lowest, lowest middle, upper middle, and highest), marital status (married or single), drinking status (never/rarely, ≤1 times/month, and >1 times/month), smoking status (non-smokers, former smoker, or current smoker), occupation (employed or not employed), day of week of dietary intake (Monday–Thursday and Friday–Saturday), regular physical activity (yes or no), body mass index (continuous, kg/m^2^), and menopausal status (yes or no, women only); Model 3 was additionally adjusted for total energy intake (continuous) and sleep duration (continuous). ^2^ Depression was defined based on self-reported doctor-diagnosed depression, and depressive symptoms were defined as feelings of sadness or desperation for ≥2 weeks during the past 1 year. ^3^ *p* values were obtained from the multivariable logistic regression models with diagnosis of depression or depressive symptoms as the outcome variable (* *p* < 0.05, ** *p* < 0.01).

## Supplementary Materials

The following are available online at https://www.mdpi.com/1660-4601/16/23/4831/s1, Table S1: The adjusted odds ratios (AORs) and 95% confidence intervals (CIs) for short or long sleep duration by night eating habits in Korean adults.
