# Supplementary material for: Association of Night Eating with Depression and Depressive Symptoms in Korean Women
Source: Int J Environ Res Public Health. 2019 Dec 1;16(23):4831. doi: 10.3390/ijerph16234831 (PMC6926998; doi:10.3390/ijerph16234831)
Supplement: Supplementary file 1 [file ijerph-16-04831-s001.pdf]

International Journal of
Environmental Research and Public Health

MDPI

Supplementary Table 1. The adjusted odds ratios (AORs) and 95% confidence intervals (CIs) for short or long sleep duration by night eating habits in Korean adults [1].

		Men			Women		
		**Non-night eaters (n = 10,890)**	**Night eaters (n = 1,830)**	***P*[2]**	**Non-night eaters (n = 17,296)**	**Night eaters (n = 1,674)**	***P***
			**AOR (95% CI)**			**AOR (95% CI)**	
Short sleep duration	Model 1	1.00	1.08 (0.96-1.22)	0.1910	1.00	1.24 (1.10-1.40)	0.0005 **
	Model 2	1.00	1.09 (0.97-1.23)	0.1336	1.00	1.19 (1.05-1.35)	0.0060 **
	Model 3	1.00	1.09 (0.97-1.23)	0.1528	1.00	1.20 (1.06-1.36)	0.0043 **
Long sleep duration	Model 1	1.00	0.99 (0.77-1.27)	0.9413	1.00	1.29 (1.05-1.59)	0.0138 *
	Model 2	1.00	0.92 (0.72-1.19)	0.5353	1.00	1.18 (0.96-1.45)	0.1269
	Model 3	1.00	0.95 (0.74-1.23)	0.7104	1.00	1.18 (0.95-1.46)	0.1278

[1] Data were from the Korea National Health and Nutrition Examination Surveys (KNHANES). All data were weighted to account for the complex study design according to the analytical guidelines of the KNHANES. AOR, adjusted odds ratio; 95% CI, 95% confidence interval. Multiple logistic regression analysis was performed to estimate the odds ratio for depression and depressive symptoms for the study participants from the KNHANES 2008–2013, in three models: The Model 1 was adjusted for age (continuous); Model 2 was additionally adjusted for education level (middle school graduates or less, high school graduates, and college graduation or higher), income (lowest, lowest middle, upper middle, and highest), marital status (married or single), drinking status (never/rarely, ≤ 1 times/month, and >1 times/month), smoking status (non-smokers, former smoker, or current smoker), occupation (employed or not employed), day of week of dietary intake (Monday–Thursday and Friday–Saturday), regular physical activity (yes or no), body mass index (continuous, kg/m^2), and menopausal status (yes or no, women only); Model 3 was additionally adjusted for total energy intake (continuous). [2] *P* values were obtained from the multivariable logistic regression models with sleep duration (short, long, or both) as the outcome variable (* $P < 0.05$, ** $P < 0.01$).
